# Innovations in Cancer Therapies and Rising Costs

**Published:** 2017-11-01

**Authors:** Pamela Hallquist Viale

It is an exciting time to be caring for patients with cancer. Great strides are being made in the treatment landscape, with new therapies approved seemingly every month. Patients have more opportunities to receive novel and innovative treatments with improved outcomes. These therapies require advanced practitioners in oncology to update and add to their current knowledge base regarding treatments for our patients, one of the important reasons to attend JADPRO Live at APSHO and stay informed!

## CAR T-CELL THERAPY APPROVED

The US Food and Drug Administration (FDA) approved a new therapy for children and young adults with B-cell acute lymphoblastic leukemia (ALL) on August 30, 2017. Chimeric antigen receptor (CAR) T-cell therapy is an important part of immunotherapy, which is emerging to become an essential part of cancer therapy. The treatment tisagenlecleucel (Kymriah) is the first-ever FDA-approved CAR T-cell therapy that genetically alters a patient’s own cells to battle the cancer itself and has the potential to change cancer therapy for the future (Grady, 2017). Approval was based on the phase II multicenter ELIANA clinical trial of 63 pediatric and young adult patients with relapsed or refractory B-cell precursor ALL; trial participants demonstrated an overall remission rate within 3 months of treatment of 83% (FDA, 2017).

The treatment involves altering the cells of each patient by removing the individual’s T cells, freezing them, and shipping the cells to a manufacturing center. The manufacturer then genetically engineers the cells, multiplies them, and ships them back to the medical center for infusion of this "living" drug into the affected patient. The treatment carries a variety of significant side effects and the potential for cytokine release syndrome, including hypotension, pulmonary edema, fever, and other life-threatening complications. Treating hospitals and clinics must be specially certified to administer the therapy per the FDA approval (FDA, 2017). And, as with all expedited or early approvals, postmarketing studies will continue to collect valuable information about the therapy and its side effects.

## RISING COSTS

It is important to mention that this innovative therapy is very expensive. The individual cost of the treatment is $475,000. The pharmaceutical company acknowledges that if a patient does not respond to treatment, there will be no charge to the individual, and that there are financial assistance programs to help defer the cost of therapy (Grady, 2017). Additionally, the company points out that alternative treatment with a bone marrow transplant is very expensive as well, ranging from $540,000 to $800,000. The very real high cost of this therapy and others adds to an uncertain health-care environment, where patients are often asked to bear a larger burden of the costs despite personal insurance.

In a recently published study, oncologist and patient discussions regarding the cost of care in breast cancer did produce positive changes in the reduction of cost (Hunter et al., 2017). Some of these cost-saving strategies involved use of different antineoplastic agents or a change in diagnostic testing for the patient. Conversations regarding cost are important in health care today, and in this study, the discussion was usually initiated by the oncologist and was fairly short, but could result in impactful information. Although there are no real medication alternatives for young patients with refractory ALL, there are potential cost savings for some therapeutic exchanges in oncology, and discussion of these options may be helpful.

The American Society of Clinical Oncology (ASCO) is committed to a discussion and framework on the rising costs of cancer care. Its value framework assesses the value of new cancer therapies, comparing clinical benefit, side effects, and potential for improvement in patient symptoms or quality of life with the cost of therapy (ASCO, 2017). The American Society of Clinical Oncology is working on a translation of the value framework into a software tool that providers can use when discussing potential therapy options with patients, empowering both providers and patients to discuss the cost of treatment and other drivers of the cost of cancer care (ASCO, 2017).

## ROLE OF ONCOLOGY ADVANCED PRACTITIONERS

Oncology advanced practitioners are directly involved in patient care and the ordering and administering of cancer therapies. The newly approved CAR T-cell therapy for pediatric patients with B-cell ALL is critically important and represents a pivotal change in our approach to these patients as well as potentially others. However, the costs of cancer care are rising, and advanced practitioners should be at the forefront of discussions regarding these costs. Whether you are sitting on your hospital’s Pharmacy and Therapeutics committee or filling out forms for financial assistance for your patient, stay involved in the discussion. The costs of cancer care are significant and can affect your patients’ quality of life as well as their pocketbooks (Hunter et al., 2017).
